# Surface electromyography characteristics of patients with anterior cruciate ligament injury in different rehabilitation phases

**DOI:** 10.3389/fphys.2023.1116452

**Published:** 2023-03-27

**Authors:** Hongxing Cui, Zhijie Cao, Shanshan Wang, Hao Zhang, Ze Chen, Xipeng Wu, Yixuan Zhao, Shuyan Qie, Wei Li

**Affiliations:** ^1^ Department of Rehabilitation, Binzhou Medical University Hospital, Binzhou, Shandong, China; ^2^ School of Rehabilitation Medicine, Binzhou Medical University, Yantai, Shandong, China; ^3^ Department of Rehabilitation, Beijing Rehabilitation Hospital, Capital Medical University, Beijing, China

**Keywords:** anterior cruciate ligament injury, surface electromyography, rehabilitation, muscle function, muscle strength

## Abstract

**Background:** Anterior cruciate ligament reconstruction (ACLR) is a common treatment for anterior cruciate ligament (ACL) injury. However, after ACLR, a significant proportion of patients do not return to pre-injury levels. Research on muscle function during movement has important implications in rehabilitation.

**Methods:** Sixty patients with unilateral ACL injury were recruited for this study and assigned into three groups: group A, individuals with an ACL injury before 6 months; group B, individuals with ACLR from 6 months to 1 year; and group C, individuals with ACLR 1 year later. Surface electromyography (SEMG) signals were collected from the bilateral rectus femoris (RF), vastus medialis (VM), vastus lateralis (VL), biceps femoris (BF), and semitendinosus (ST). The tasks performed during the experiment included straight leg raising (SLR) training at 30°, SLR training at 60°, ankle dorsiflexion, walking, and fast walking.

**Results:** In the maximum muscle strength test, the affected side of the BF in group A (199.4 ± 177.12) was significantly larger than in group B (53.91 ± 36.61, *p* = 0.02) and group C (75.08 ± 59.7, *p* = 0.023). In the walking test, the contralateral side of the RF in group B (347.53 ± 518.88) was significantly greater than that in group C (139.28 ± 173.78, *p* = 0.029). In the SLR training (60°) test, the contralateral side of the RF in group C (165.37 ± 183.06) was significantly larger than that in group A (115.09 ± 62.47, *p* = 0.023) and smaller than that in group B (226.21 ± 237.17, *p* = 0.046); In the ankle dorsiflexion training test, the contralateral side of the RF in group B (80.37 ± 87.9) was significantly larger than that in group C (45.61 ± 37.93, *p* = 0.046).

**Conclusion:** This study showed the EMG characteristics of patients with ACL injury helped to determine which muscle requires more training and which exercise model would be best suited for intervention.

## 1 Introduction

Anterior cruciate ligament reconstruction (ACLR) is a common treatment option for patients with anterior cruciate ligament (ACL) injuries (Van Melick et al., 2016). It is estimated that up to 200,000 ACLRs are performed annually in the United States (Fryer et al., 2019). Patient success rate for primary ACLR is approximately 75%–97% (Kim et al., 2018). However, after ACLR, a significant proportion of patients do not return to their pre-injury levels. A systematic review and meta-analysis by Ardern et al. showed that 69 studies found 81% of individuals reported returning to sports after ACLR. In contrast, only 65% reported returning to the pre-injury level of sports participation (Ardern et al., 2014). Recent research shows that 35% of athletes do not return to the pre-injury sports level within 2 years after ACLR, and half reported their ACL injury as the primary reason for a lower activity level (Ardern et al., 2011; Ardern et al., 2012; Ardern et al., 2014). The prevalence of these adverse outcomes underscores the importance of rehabilitation after ACLR, so further research is warranted on how to improve the effectiveness of rehabilitation.

In addition to restoring joint stability, ACLR surgery aims to restore knee joint function and muscle strength (Kim et al., 2015). An appropriate rehabilitation program was incorporated into routine preoperative and postoperative care to maximize surgical outcomes and improve functional recovery. Impaired knee function associated with ACL injury includes instability in dynamic movements and weakness of the quadriceps (Kim et al., 2018). Quadriceps muscle weakness, critical to dynamic joint stability, eventually leads to decreased knee function and poor exercise performance and can contribute to the early onset of osteoarthritis (Muaidi et al., 2007). Thus, the function of the muscles around the knee joint, especially the quadriceps femoris (QF), is the main training target and serves as an indicator for monitoring functional recovery after ACLR surgery (Slemenda et al., 1997). After ACLR, most patients have lower extremity muscle strength deficits on the affected and contralateral sides. Specifically, between-limb QF muscle strength symmetry is recommended as an important clinical benchmark to determine whether an athlete is ready for sports after ACLR (Logerstedt et al., 2017).

An increasing number of studies have focused on evaluating the primary ACLR surgery method, joint stability, muscle strength, and knee function (Muaidi et al., 2007; Kim et al., 2015). Jong et al. reported that lower extremity muscle atrophy and weakness after ACLR represent a difficult and unresolved problem (De Jong et al., 2007). Unresolved post-operative muscle strength deficits might be associated with knee osteoarthritis that is present years after surgery (Keays et al., 2010). Investigators have also reported post-operative strength deficits ranging from 5% to 40% for the quadriceps and from 9% to 27% for the hamstrings (De Jong et al., 2007; Hiemstra et al., 2007; Karanikas et al., 2009). However, limited studies have assessed muscle strength recovery after revision ACLR surgery during different sports modes.

Limited recovery of lower extremity muscle function may be related to the current rehabilitation program. Most studies have described current time-based rehabilitation protocols that are mainly based on the remodeling process of the graft (Czuppon et al., 2014). However, there is still uncertainty regarding the schedule of the human remodeling process, and more experts are considering incorporating functional goal-based criteria into the rehabilitation protocol (Howells et al., 2011). In addition, there were individual differences in neuromotor learning and flexibility after ACLR. These findings underscore the importance of a change from time-based rehabilitation to goal-based rehabilitation with neuromuscular goals and criteria to manage the rehabilitation process. Österberg et al. (2013) suggested that these goals for progression to the next phase and description of interventions during each phase should be based on the International Classification of Functioning, Disability, and Health, which may be more suitable for the rehabilitation of patients. Among these studies on rehabilitation after ACLR, few studies have focused on the evaluation of quality of movement. However, the relevance of focusing more on the quality of movement is underlined by the fact that altered neuromuscular function and biomechanics after ACLR could be risk factors for a second ACL injury (Hiemstra et al., 2009).

Surface electromyography (SEMG) has the advantages of non-invasion, real-time, and multitarget measurement, and is a method that has received increasing attention due to its ability to quantitatively analyze neuromuscular activity in static and dynamic motion states (Rasool et al., 2017). SEMG has been used to assess normal and abnormal muscle activation in some patients with ACL injury to guide rehabilitation strategies (Zebis et al., 2019). Nevertheless, it is rarely used to assess muscle activation at different stages of rehabilitation.

After ACL injury and reconstruction, lower extremity muscle strength has been reported to decrease not only in the quadriceps but also in the hamstrings, usually continuing after the postoperative rehabilitation period. In vigorous dynamic movements, coactivation of the hamstring tendons is important to provide dynamic stability of the knee and prevent excessive shear forces of the ACL. Kim et al. found that after an ACL injury and subsequent reconstruction, deficits in quadriceps muscle strength were significant (Kim et al., 2015). Bade et al. showed that in early high intensity and low intensity rehabilitation after total knee, quadriceps and hamstring strength and quadriceps activation improved beyond baseline performance in both groups (Bade et al., 2017). Thus, we performed this study to assess lower muscle functional recovery before and after ACLR surgery at different periods using different sports modes. We aimed to 1) compare the recovery of knee extensor muscle function (rectus femoris [RF], vastus medialis [VM], vastus lateralis [VL]) and flexor muscle (biceps femoris [BF] and semitendinosus [ST]) before and after ACLR and 2) compare the functional outcomes of different sports modes. We hypothesized that patients with ACL injury would exhibit different characteristics of SEMG in different rehabilitation phases and in different training tasks.

## 2 Methods

### 2.1 Recruitment of participants

In this study, based on literature reports and previous research data, using G*Power software (3.1.9.7) calculated sample size, sixty patients with unilateral ACL injuries were recruited. Patients who met the following criteria were included: 1) age >18 years; 2) diagnosed as ACL injury by magnetic resonance imaging and clinical examination or had received ACLR surgery using the all-soft tissue quadriceps tendon. The excluded criteria were the following: 1) patients with previous ACLR surgery and recurrence of ACL injury. 2) patients with cognitive impairment unable to complete experimental procedures; 3) patients with a history of other neurological diseases or disorders, lower extremity surgery, or fracture. Patients eligible for enrolment were divided into three groups according to the time of injury and the time to surgery. All patients completed routine preoperative rehabilitation. Individuals with ACL injury less than half a year were recruited as Group A (16 men and 4 women, average age: 33.5 ± 11.77 years; height: 171 ± 5.36 cm; weight: 70.1 ± 13.71 kg); Individuals with ACLR for less than half a year were recruited as Group B (13 men and 7 women, average age: 32.35 ± 9.4 years; height: 171.85 ± 8.46 cm; weight: 75.98 ± 17.49 kg); Individuals with ACLR more than half a year and less than 1 year were recruited as Group C (14 men and 6 women, average age: 31.45 ± 8.02 years; height: 171.35 ± 7.81 cm; weight: 71.88 ± 12.25 kg). Patients with knee disorders, anatomical abnormalities, or a history of surgery were excluded. The Medical Ethics Committee of Binzhou Medical University Hospital approved this study (2021-S019-01). A signed consent form was obtained from each participant before the test was performed.

### 2.2 Measurement process

**FIGURE 1:**
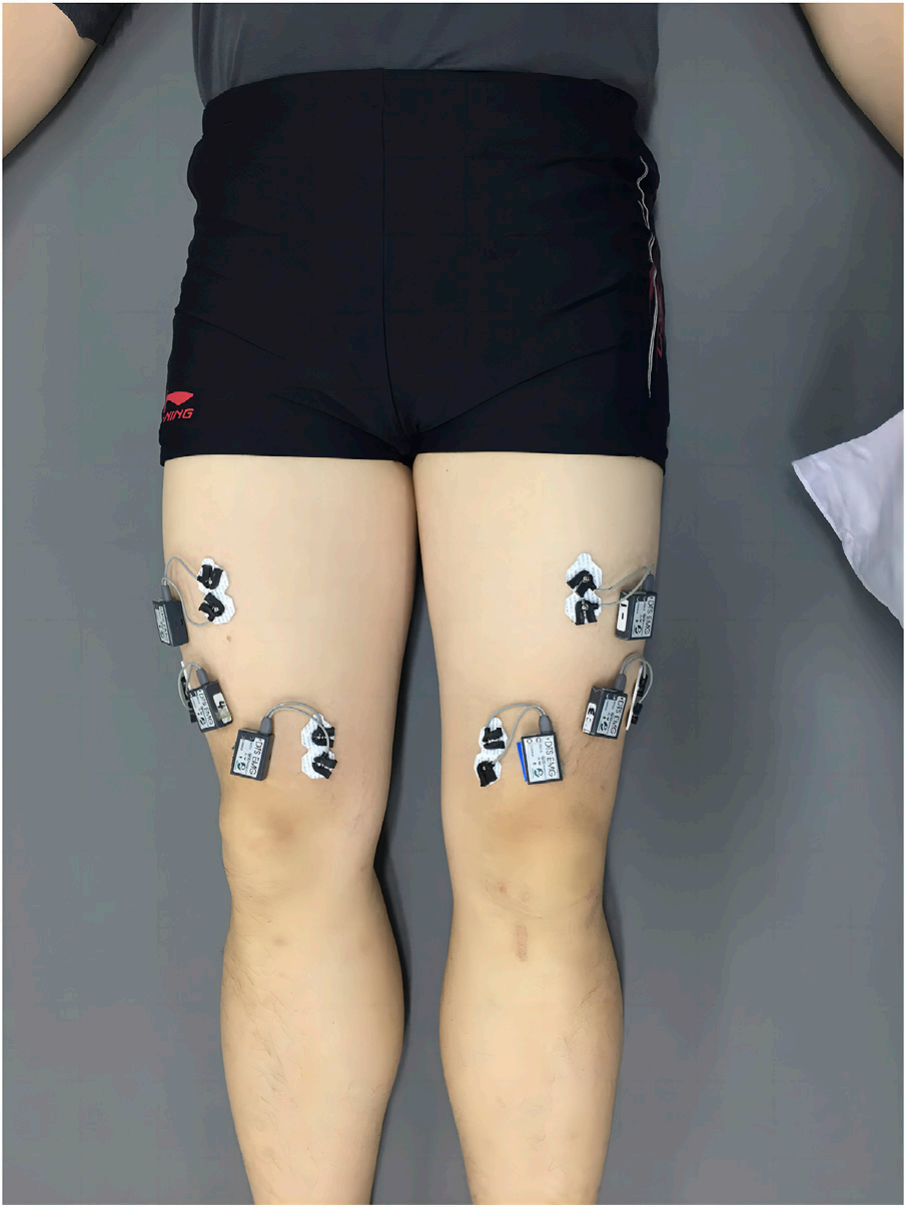
Electrode positions are shown in the Figure.

Surface electromyography (SEMG) was performed using a Noraxon wireless dynamic electromyography tester (Noraxon, Scottsdale, AZ, USA). The sampling frequency was 1,200 Hz. After scraping, abrading, and alcohol cleaning, the electrodes were fixed onto the patient’s skin (Figure 1). SEMG signals were collected from the RF, VM, VL, BF, and ST. The signals were sampled at 1,200 Hz with a bandpass filter of 20–500 Hz. The root mean square (RMS) was used to assess muscular activity. The tasks performed during the experiment included straight leg raising (SLR) training at 30°, SLR training at 60°, ankle dorsiflexion, walking, and fast walking.

### 2.3 Data collection

Before the experiment began, we explained in detail what the participants needed to accomplish. Maintaining the stability of the knee while spinning is an important function of ACL (Markatos et al., 2013; Van Melick, et al., 2022); thus, we chose it as a walking task. The patient walked continuously on an elliptical curve for 5 min at the most comfortable speed. The width of the examination room was 6 meters, and the length was 8 meters. We separately acquire bilateral lower limbs EMG signals. And the middle 3 min EMG signal was used for analysis during the walking process. Similarly, when walking quickly, the patient was required to walk for 5 min at the fastest speed and the analysis test process was carried out for 3 min in the middle. The patient laid supine on the bed, baseline SEMG signals were measured during a state of muscle relaxation, then raised the leg 30° or 60° and dorsiflexed of the ankle, and maintained this position for 10 s (Li et al., 2020; Mitchell et al., 2022; Molina-Cárdenas et al., 2022). The experiment was repeated three times. The test is considered successful to see if the SEMG signal increases to more than 3 times the baseline signal with the active contraction movement of the muscle. The above tests are performed first on the contralateral side and then on the affected side. In the test of the maximum muscle strength of the quadriceps femoris, the patient performs a knee extension exercise in the supine position, keeping the calf hanging at the end of the bed and applying resistance at the lower end of the calf. The maximum muscle strength of the BF and ST was applied by having the patient bend the knee in the prone position and apply resistance to the crus (Dixit et al., 2022). The movement of the maximum muscle strength test is also a training exercise in the rehabilitation program. We performed an sEMG signal test on the muscle surface at maximum muscle strength to assess muscle activation during the movement, which was useful for us to evaluate the effectiveness of the activity in the rehabilitation program. The doctors and nurses accompanied the patients to ensure safety during the test.

### 2.4 Data process

SEMG signals were processed and analyzed using EMGworks Analysis and MATLAB R2020a (MathWorks, 3 Apple Hill Dr, Natick, MA 01760-2098). The sampling frequency was 1,200 Hz. The root mean square (RMS) value was analyzed and used to evaluate muscle activity.

### 2.5 Statistical methods

All data were analyzed using SPSS software (version 19.0). Data are presented as mean ± standard deviation (M±SD). The Shapiro–Wilk normality test was performed to examine the normality distribution of the data. One-way ANOVA and post-hoc Least-Significant Difference tests were used to detect statistically significant differences of the variables. Statistical significance was established at *p* < 0.05.

## 3 Results

**FIGURE 2:**
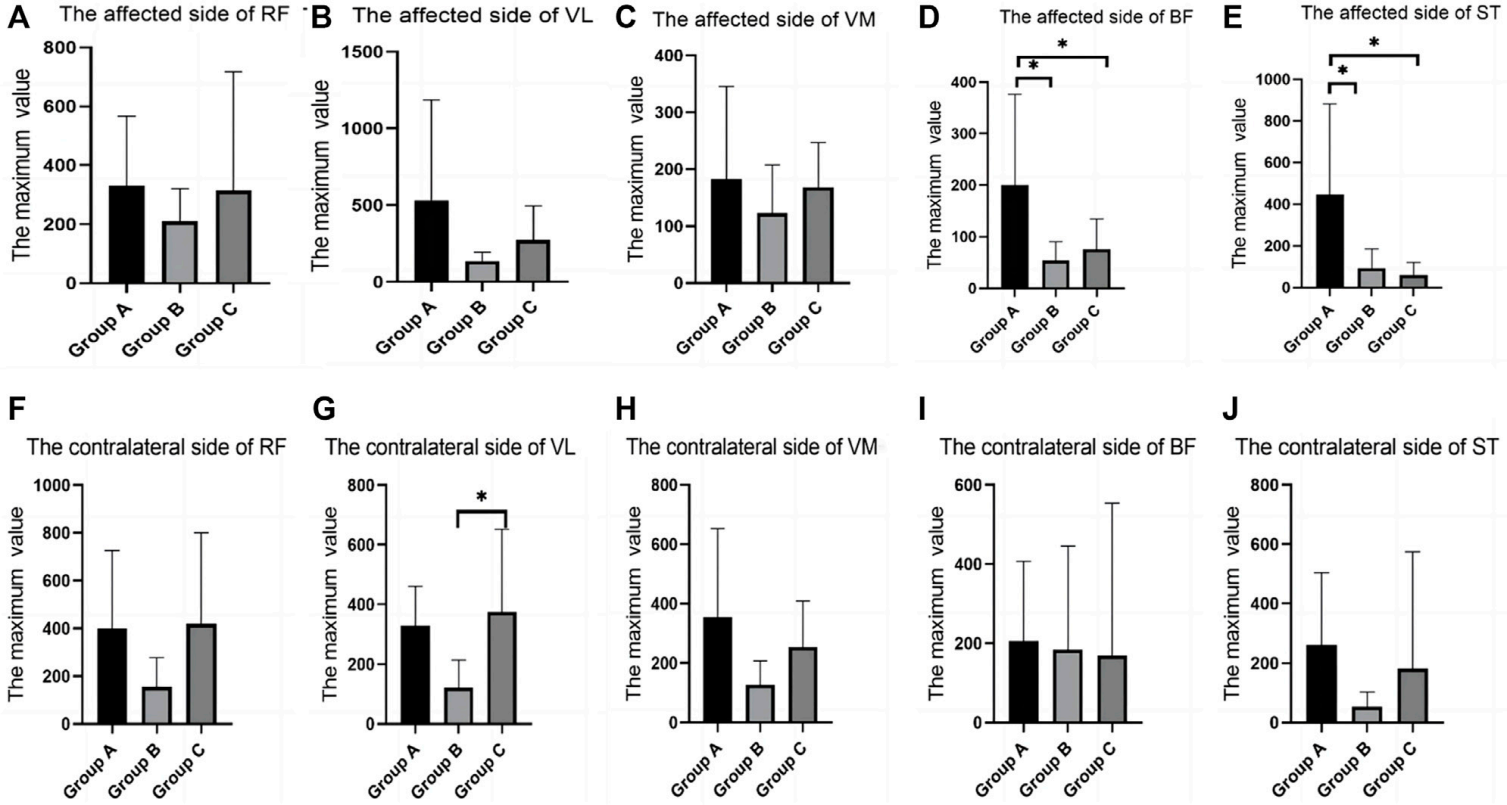
SEMG values of maximum muscle strength test between different groups. **(A)**, The affected side of RF; **(B)**, The affected side of VL; **(C)**, The affected side of VM; **(D)**, The affected side of BF; **(E)**, The affected side of ST; **(F)**, The contralateral side of RF; **(G)**, The contralateral side of VL; **(H)**, The contralateral side of VM; **(I)**, The contralateral side of BF; **(J)**, The contralateral side of ST; SEMG, surface electromyography; RF, rectus femoris; VM, vastus medialis; VL, vastus lateralis; BF, biceps femoris; ST, semitendinosus.

In the maximum strength test, there were differences between groups in the RMS values of BF and ST on the affected side, and group A was significantly higher than group B and Group C (Table 1; Figure 2).

**TABLE 1:** SEMG values of maximum muscle strength test between different groups.

	Groups	Mean ± SD	Groups		P-value
Affected side -RF	A	329.98 ± 236.46	A	B	0.515
	B	210.03 ± 110.6	A	C	0.924
	C	314.63 ± 402.42	B	C	0.518
Contralateral side -RF	A	400.3 ± 326.11	A	B	0.201
	B	155.27 ± 122.3	A	C	0.916
	C	417.73 ± 381.76	B	C	0.123
Affected side -VL	A	531.33 ± 653.88	A	B	0.074
	B	134.77 ± 58.04	A	C	0.176
	C	272.36 ± 223.1	B	C	0.465
Contralateral side -VL	A	329.33 ± 130.88	A	B	0.104
	B	121.21 ± 92.71	A	C	0.674
	C	375.18 ± 277.15	B	C	**0.028**
Affected side -VM	A	182.3 ± 163.28	A	B	0.347
	B	122.5 ± 84.99	A	C	0.798
	C	168.13 ± 78.7	B	C	0.413
Contralateral side -VM	A	353.63 ± 299.69	A	B	0.053
	B	127.25 ± 81.33	A	C	0.312
	C	253.36 ± 155.73	B	C	0.207
Affected side -BF	A	199.4 ± 177.12	A	B	**0.02**
	B	53.91 ± 36.61	A	C	**0.023**
	C	75.08 ± 59.7	B	C	0.681
Contralateral side -BF	A	205.18 ± 201.7	A	B	0.91
	B	184.1 ± 260.93	A	C	0.825
	C	169.05 ± 384.54	B	C	0.927
Affected side -ST	A	446.4 ± 435.73	A	B	**0.014**
	B	93.25 ± 91.91	A	C	**0.003**
	C	59.07 ± 61.64	B	C	0.77
Contralateral side -ST	A	260.42 ± 243.44	A	B	0.252
	B	53.05 ± 49.4	A	C	0.613
	C	181.01 ± 393.31	B	C	0.418

SEMG, surface electromyography; RF, rectus femoris; VM, vastus medialis; VL, vastus lateralis; BF, biceps femoris; ST, semitendinosus.

That the bold values indicates the statistical significance was established.

**FIGURE 3:**
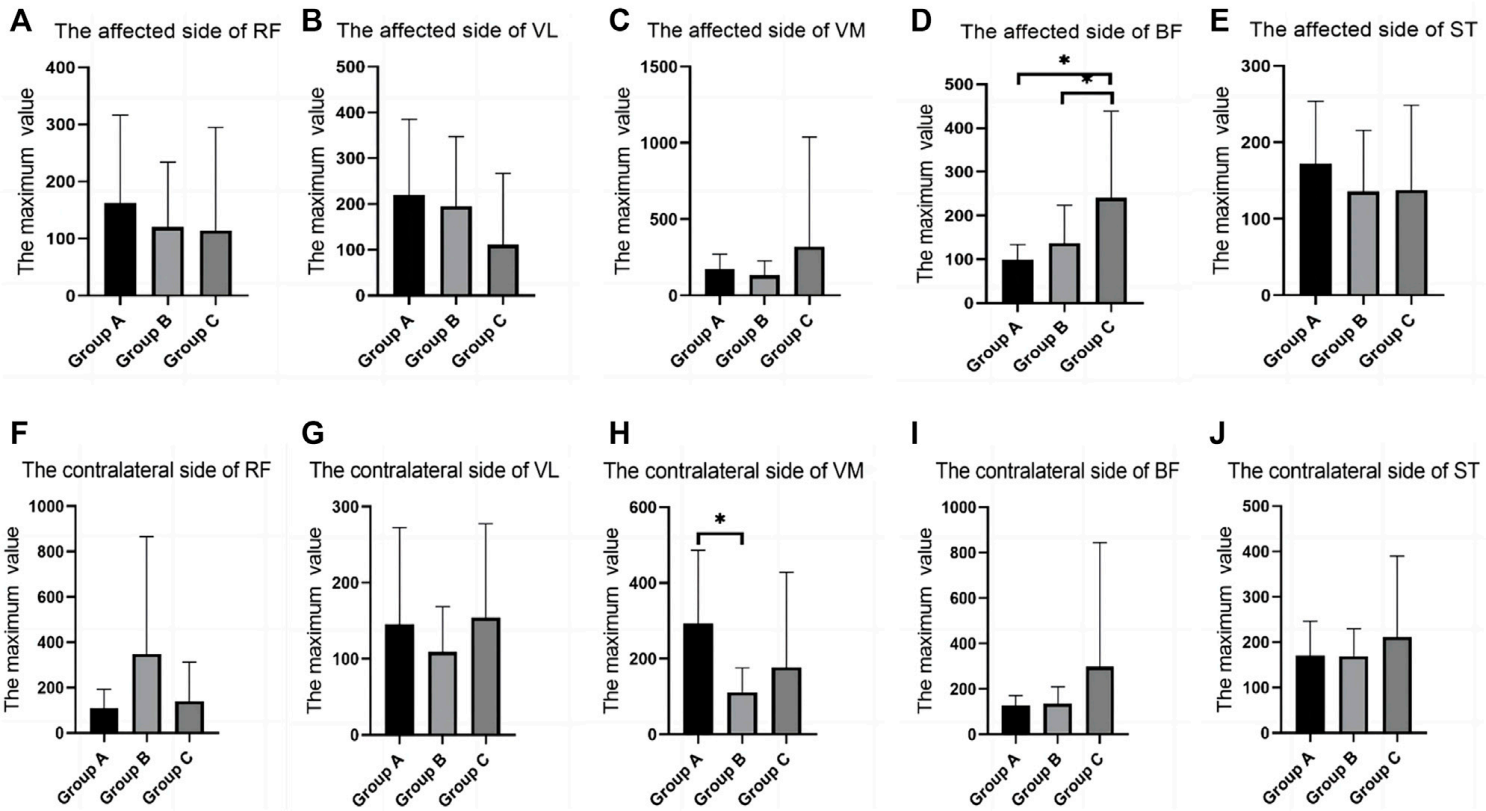
SEMG values of different groups during walking. **(A)**, The affected side of RF; **(B)**, The affected side of VL; **(C)**, The affected side of VM; **(D)**, The affected side of BF; **(E)**, The affected side of ST; **(F)**, The contralateral side of RF; **(G)**, The contralateral side of VL; **(H)**, The contralateral side of VM; **(I)**, The contralateral side of BF; **(J)**, The contralateral side of ST; SEMG, surface electromyography; RF, rectus femoris; VM, vastus medialis; VL, vastus lateralis; BF, biceps femoris; ST, semitendinosus.

In the walking test, there were differences between groups in the RMS values of RF, VM, and BF. The RF of the contralateral side in group B was significantly higher than that in group C; the VM of the contralateral side in group A was significantly higher than that in group B. The BF of the affected side in group C was significantly higher than that in group A and group B (Table 2; Figure 3).

**TABLE 2:** SEMG values of different groups during walking.

	Groups	Mean ± SD	Groups		P-value
Affected side -RF	A	162.16 ± 154.37	A	B	0.527
	B	120.09 ± 114.12	A	C	0.416
	C	114.09 ± 181.05	B	C	0.906
Contralateral side -RF	A	109.58 ± 82.28	A	B	0.055
	B	347.53 ± 518.88	A	C	0.784
	C	139.28 ± 173.78	B	C	**0.029**
Affected Side -VL	A	219.27 ± 166.07	A	B	0.705
	B	194.95 ± 152.2	A	C	0.062
	C	111.09 ± 155.7	B	C	0.093
Contralateral side -VL	A	145.72 ± 126.89	A	B	0.423
	B	109.09 ± 59.27	A	C	0.833
	C	154.26 ± 123.45	B	C	0.199
Affected side -VM	A	173.25 ± 96.18	A	B	0.853
	B	131.67 ± 95.65	A	C	0.458
	C	321.24 ± 717.19	B	C	0.273
Contralateral Side -VM	A	292.86 ± 192.94	A	B	**0.038**
	B	111.61 ± 64.62	A	C	0.133
	C	177.78 ± 250.88	B	C	0.315
Affected Side -BF	A	99.45 ± 34.28	A	B	0.561
	B	137.07 ± 86.54	A	C	**0.017**
	C	240.03 ± 198.78	B	C	**0.042**
Contralateral side -BF	A	127.68 ± 43.97	A	B	0.958
	B	136.57 ± 72.78	A	C	0.264
	C	297.65 ± 546.23	B	C	0.221
Affected side -ST	A	172.25 ± 81.63	A	B	0.368
	B	135.52 ± 80.08	A	C	0.34
	C	137.63 ± 110.93	B	C	0.946
Contralateral side -ST	A	169.74 ± 76.27	A	B	0.997
	B	169.55 ± 60.07	A	C	0.423
	C	211.25 ± 178.38	B	C	0.352

SEMG, surface electromyography; RF, rectus femoris; VM, vastus medialis; VL, vastus lateralis; BF, biceps femoris; ST, semitendinosus.

That the bold values indicates the statistical significance was established.

**FIGURE 4:**
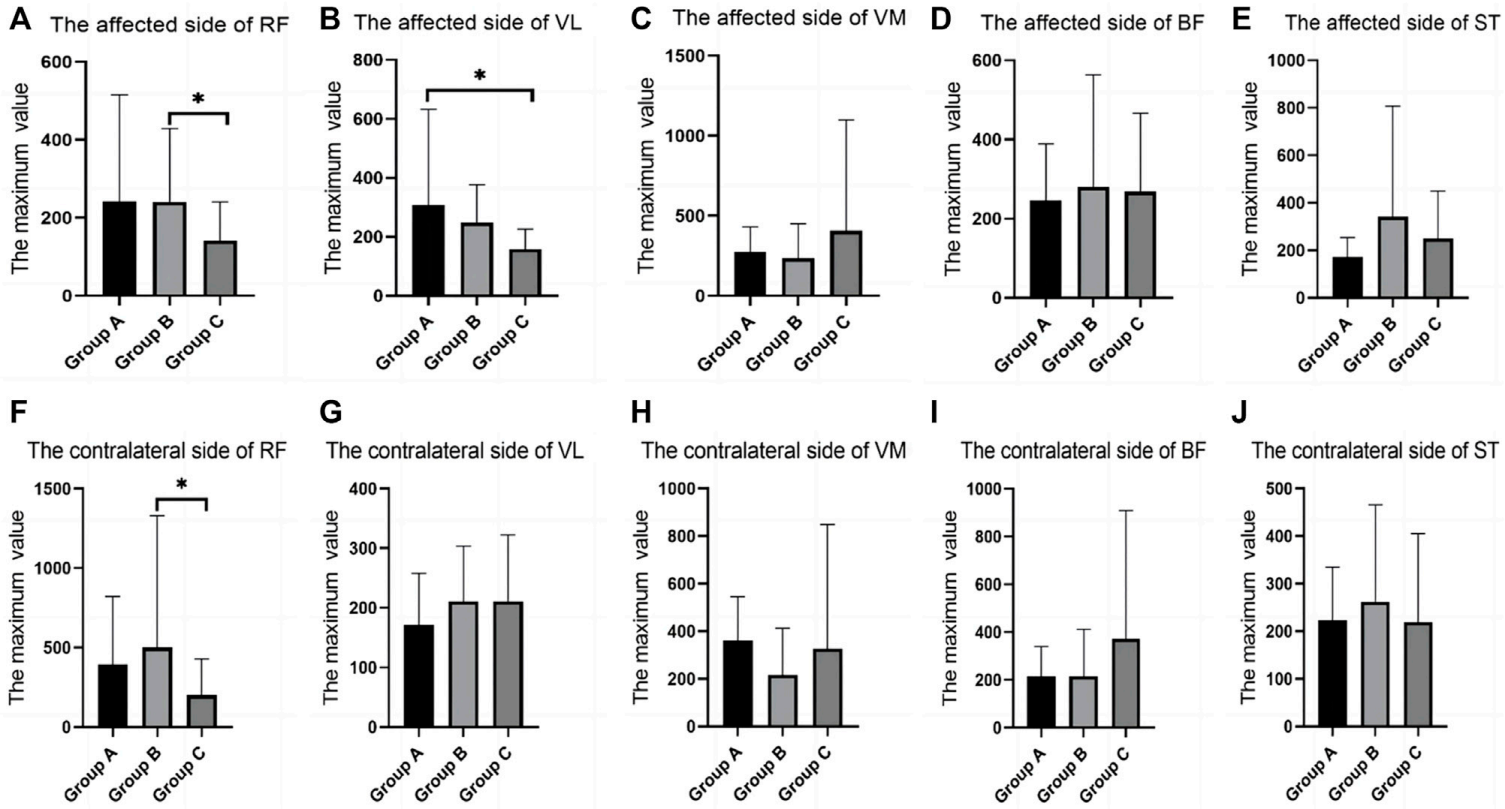
SEMG values during fast walking. **(A)**, The affected side of RF; **(B)**, The affected side of VL; **(C)**, The affected side of VM; **(D)**, The affected side of BF; **(E)**, The affected side of ST; **(F)**, The contralateral side of RF; **(G)**, The contralateral side of VL; **(H)**, The contralateral side of VM; **(I)**, The contralateral side of BF; **(J)**, The contralateral side of ST; SEMG, surface electromyography; RF, rectus femoris; VM, vastus medialis; VL, vastus lateralis; BF, biceps femoris; ST, semitendinosus.

In the fast-walking test, there were differences between groups in the RMS values of RF and VL. The RF of both sides in group B were significantly higher than that in group C. The VL of the affected side in group A was significantly higher than that in group C (Table 3; Figure 4).

**TABLE 3:** SEMG values during fast walking.

	Groups	Mean ± SD	Groups		P-value
Affected side -RF	A	241.22 ± 274.33	A	B	0.993
	B	240.71 ± 188.02	A	C	0.079
	C	141.08 ± 99.02	B	C	**0.044**
Contralateral side -RF	A	391.95 ± 430.19	A	B	0.557
	B	500.38 ± 828.46	A	C	0.257
	C	199.96 ± 227.7	B	C	**0.042**
Affected side -VL	A	306.77 ± 324.57	A	B	0.324
	B	248.57 ± 128.09	A	C	**0.007**
	C	158.12 ± 67.57	B	C	0.055
Contralateral side -VL	A	171.33 ± 86.88	A	B	0.281
	B	210.73 ± 92.93	A	C	0.233
	C	211.24 ± 111.1	B	C	0.986
Affected side -VM	A	276.23 ± 154.71	A	B	0.825
	B	235.61 ± 214.4	A	C	0.44
	C	406.61 ± 692.31	B	C	0.243
Contralateral side -VM	A	362.23 ± 182.44	A	B	0.31
	B	217.02 ± 195.37	A	C	0.783
	C	326.33 ± 522.64	B	C	0.333
Affected side -BF	A	245.77 ± 143.18	A	B	0.661
	B	280.05 ± 282.65	A	C	0.756
	C	267.97 ± 198	B	C	0.845
Contralateral side -BF	A	215.38 ± 125.05	A	B	0.924
	B	215.38 ± 196.95	A	C	0.24
	C	371.69 ± 537.51	B	C	0.175
Affected side -ST	A	171.4 ± 81.42	A	B	0.108
	B	341.97 ± 465.56	A	C	0.417
	C	249.48 ± 199.2	B	C	0.267
Contralateral side -ST	A	222.96 ± 111.68	A	B	0.547
	B	262.21 ± 203.31	A	C	0.945
	C	218.91 ± 186.31	B	C	0.404

SEMG, surface electromyography; RF, rectus femoris; VM, vastus medialis; VL, vastus lateralis; BF, biceps femoris; ST, semitendinosus.

That the bold values indicates the statistical significance was established.

**FIGURE 5:**
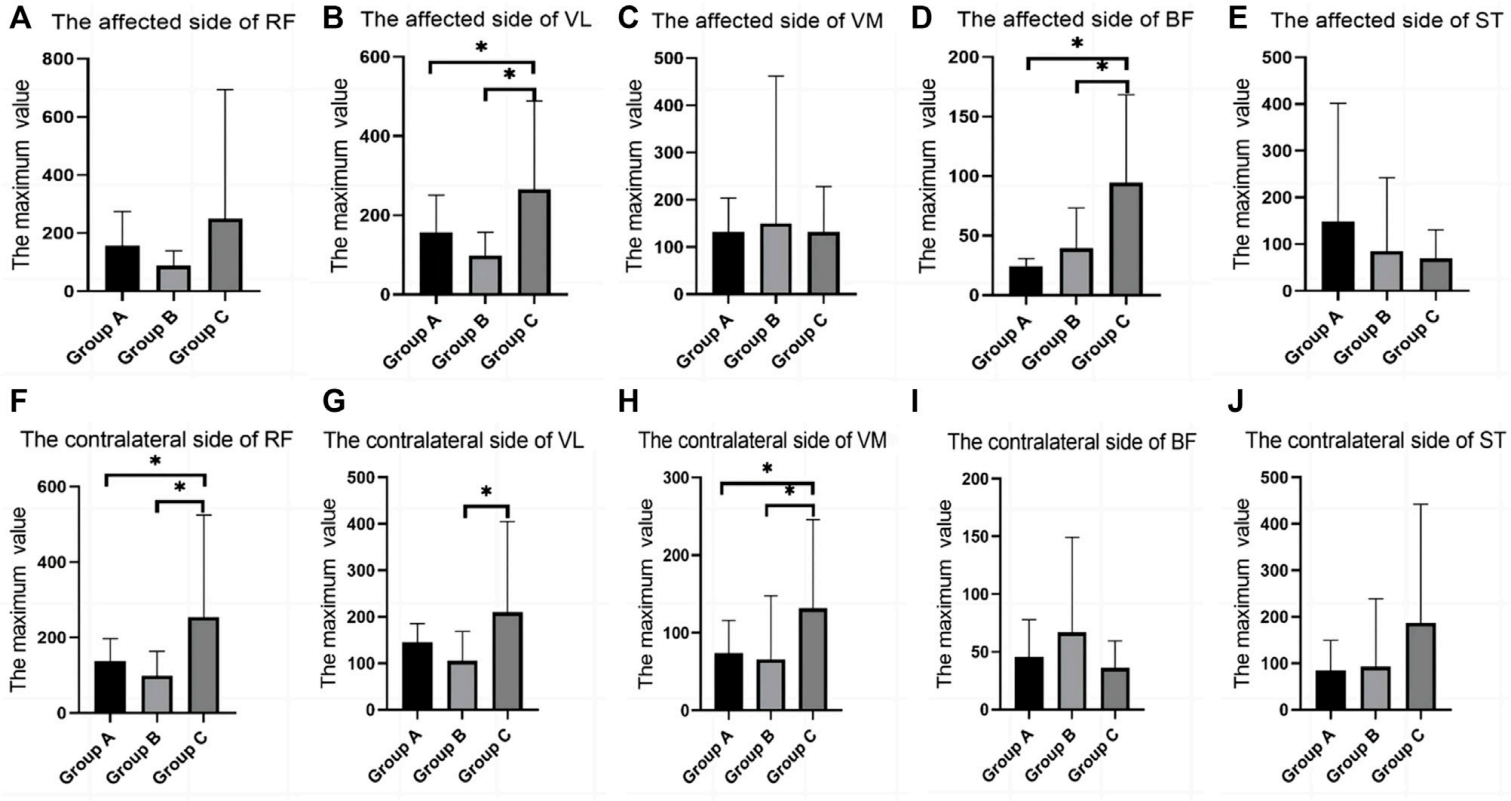
SEMG values during straight leg raising training (30°). **(A)**, The affected side of RF; **(B)**, The affected side of VL; **(C)**, The affected side of VM; **(D)**, The affected side of BF; **(E)**, The affected side of ST; **(F)**, The contralateral side of RF; **(G)**, The contralateral side of VL; **(H)**, The contralateral side of VM; **(I)**, The contralateral side of BF; **(J)**, The contralateral side of ST; SEMG, surface electromyography; RF, rectus femoris; VM, vastus medialis; VL, vastus lateralis; BF, biceps femoris; ST, semitendinosus.

In the SLR training (30°) test, there were differences between groups in the RMS values of the RF, VL, VM and BF. RMS values of Group C was significantly higher than group A and Group B. VL of the contralateral side of VL in group B was significantly decreased when compared with that in group C (Table 4; Figure 5).

**TABLE 4:** SEMG values during straight leg raising training (30°).

	Groups	Mean ± SD	Groups		P-value
Affected side -RF	A	157.88 ± 116.82	A	B	0.495
	B	87.37 ± 51.52	A	C	0.34
	C	249.72 ± 444.73	B	C	0.078
Contralateral side -RF	A	137.04 ± 60.52	A	B	0.546
	B	98.91 ± 65.06	A	C	**0.049**
	C	254.45 ± 269.72	B	C	**0.007**
Affected side -VL	A	157.11 ± 93.6	A	B	0.28
	B	98.22 ± 58.74	A	C	**0.036**
	C	264.84 ± 223.52	B	C	**0.001**
Contralateral side -VL	A	144.71 ± 40.56	A	B	0.39
	B	104.77 ± 63.17	A	C	0.137
	C	209.4 ± 195.43	B	C	**0.013**
Affected side -VM	A	131.68 ± 71.9	A	B	0.777
	B	149.79 ± 311.9	A	C	0.997
	C	131.88 ± 96.41	B	C	0.751
Contralateral side -VM	A	73.94 ± 41.95	A	B	0.794
	B	65.86 ± 81.77	A	C	**0.049**
	C	131.37 ± 114.17	B	C	**0.019**
Affected side -BF	A	24.36 ± 6.32	A	B	0.401
	B	39.43 ± 33.75	A	C	**0.001**
	C	94.44 ± 73.88	B	C	**0.001**
Contralateral side -BF	A	45.18 ± 32.69	A	B	0.22
	B	66.56 ± 82.5	A	C	0.575
	C	36.13 ± 23.32	B	C	0.051
Affected side -ST	A	148.64 ± 252.89	A	B	0.244
	B	84.9 ± 157.34	A	C	0.121
	C	69.38 ± 61.13	B	C	0.746
Contralateral side -ST	A	84.97 ± 64.26	A	B	0.909
	B	92.37 ± 146.48	A	C	0.096
	C	186.66 ± 255.54	B	C	0.104

SEMG, surface electromyography; RF, rectus femoris; VM, vastus medialis; VL, vastus lateralis; BF, biceps femoris; ST, semitendinosus.

That the bold values indicates the statistical significance was established.

**FIGURE 6:**
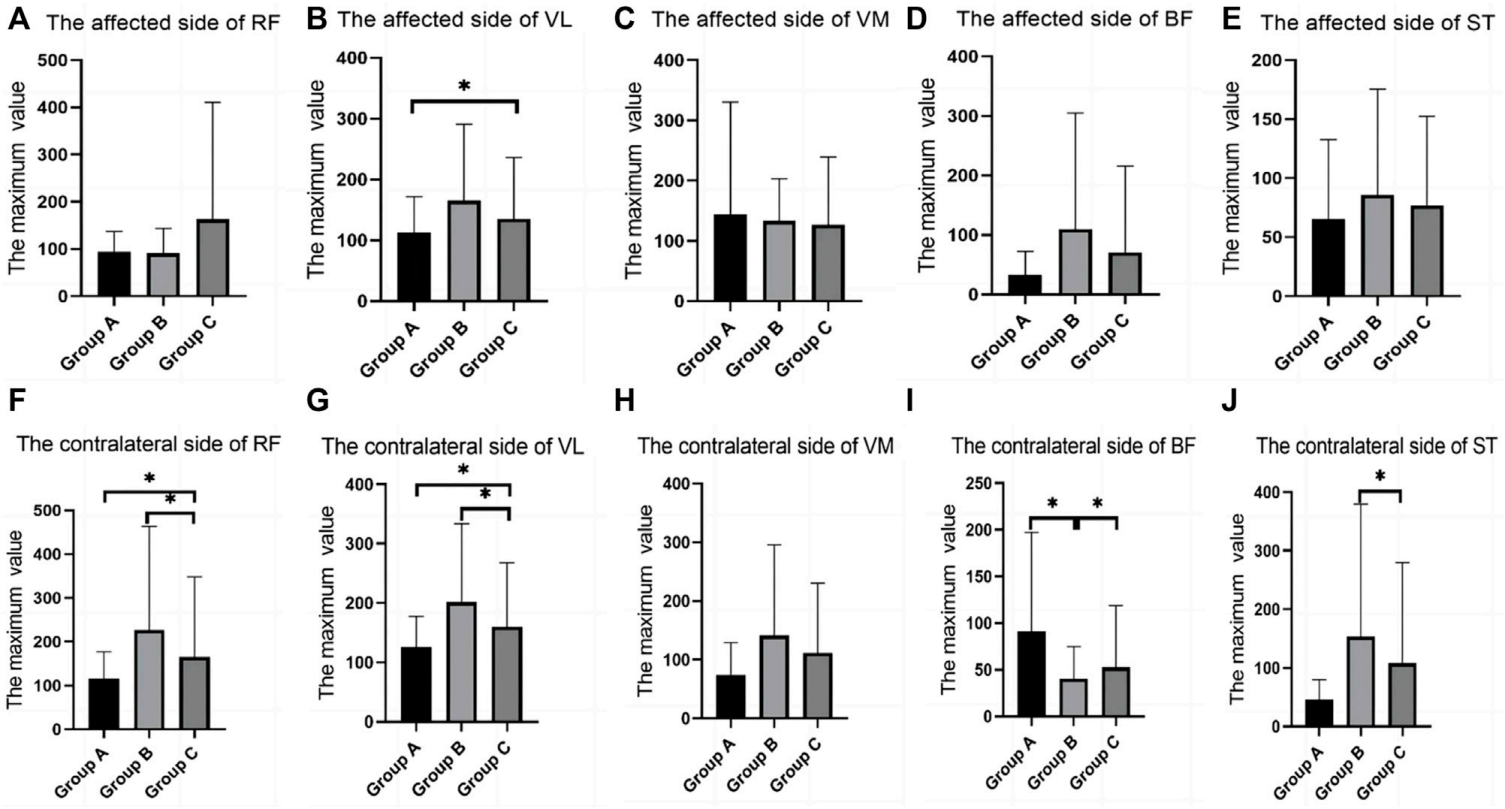
The SEMG values during straight leg raising training (60°). **(A)**, The affected side of RF; **(B)**, The affected side of VL; **(C)**, The affected side of VM; **(D)**, The affected side of BF; **(E)**, The affected side of ST; **(F)**, The contralateral side of RF; **(G)**, The contralateral side of VL; **(H)**, The contralateral side of VM; **(I)**, The contralateral side of BF; **(J)**, The contralateral side of ST; SEMG, surface electromyography; RF, rectus femoris; VM, vastus medialis; VL, vastus lateralis; BF, biceps femoris; ST, semitendinosus.

In the SLR training (60°) test, there were differences in RMS values for RF, VL, BF, and ST between groups. The contralateral RF in group C was shown to be significantly larger than that in group A and smaller than that in group B. The contralateral VL in group C was significantly larger than that in group A and smaller than that in group B. The VL of the affected side in group A was significantly decreased when compared with that in group C. The ST of contralateral side in group B was significantly larger than that in group C. The VL of the affected side in group A was significantly decreased when compared with that in group C. The contralateral BF in group B was significantly decreased when compared with that in group A and group C (Table 5; Figure 6).

**TABLE 5:** The SEMG values during straight leg raising training (60°).

	Groups	Mean ± SD	Groups		P-value
Affected side -RF	A	94.67 ± 42.27	A	B	0.959
	B	91.06 ± 52.67	A	C	0.277
	C	163.19 ± 247.96	B	C	0.208
Contralateral side -RF	A	115.09 ± 62.47	A	B	0.658
	B	226.21 ± 237.17	A	C	**0.023**
	C	165.37 ± 183.06	B	C	**0.046**
Affected side -VL	A	113.1 ± 58.84	A	B	0.586
	B	165.13 ± 125.92	A	C	**0.035**
	C	135.28 ± 100.54	B	C	0.092
Contralateral side -VL	A	126.08 ± 51.23	A	B	0.566
	B	201.71 ± 131.8	A	C	**0.006**
	C	159.89 ± 107.75	B	C	**0.019**
Affected side -VM	A	144.16 ± 186.39	A	B	0.186
	B	133.62 ± 69.74	A	C	0.229
	C	126.64 ± 112.94	B	C	0.766
Contralateral side -VM	A	74.36 ± 54.74	A	B	0.733
	B	140.77 ± 154.8	A	C	0.208
	C	111.37 ± 118.88	B	C	0.074
Affected side -BF	A	32.92 ± 39.15	A	B	0.908
	B	109.87 ± 194.94	A	C	0.094
	C	70.75 ± 145.23	B	C	0.088
Contralateral side -BF	A	90.96 ± 106.28	A	B	**0.017**
	B	40.21 ± 34.47	A	C	0.695
	C	52.68 ± 66.19	B	C	**0.012**
Affected side -ST	A	65.13 ± 67.45	A	B	0.826
	B	85.7 ± 89.89	A	C	0.593
	C	76.95 ± 75.57	B	C	0.391
Contralateral side -ST	A	45.9 ± 33.91	A	B	0.579
	B	153.91 ± 225.5	A	C	0.214
	C	108.37 ± 171.34	B	C	**0.043**

SEMG, surface electromyography; RF, rectus femoris; VM, vastus medialis; VL, vastus lateralis; BF, biceps femoris; ST, semitendinosus.

That the bold values indicates the statistical significance was established.

**FIGURE 7:**
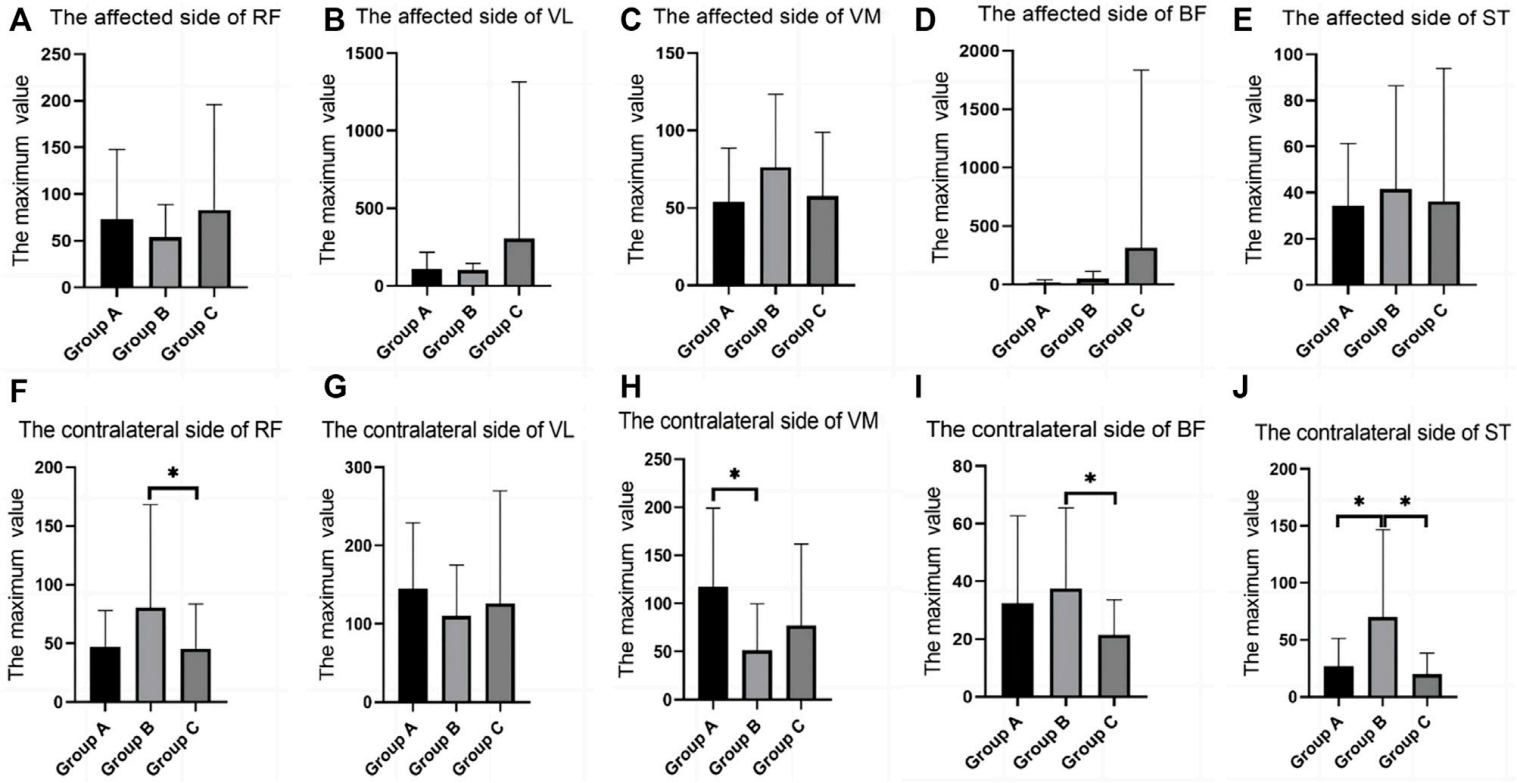
SEMG values during ankle dorsiflflexion training. **(A)**, The affected side of RF; **(B)**, The affected side of VL; **(C)**, The affected side of VM; **(D)**, The affected side of BF; **(E)**, The affected side of ST; **(F)**, The contralateral side of RF; **(G)**, The contralateral side of VL; **(H)**, The contralateral side of VM; **(I)**, The contralateral side of BF; **(J)**, The contralateral side of ST; SEMG, surface electromyography; RF, rectus femoris; VM, vastus medialis; VL, vastus lateralis; BF, biceps femoris; ST, semitendinosus.

In the ankle dorsiflexion training test, there were differences between groups in the RMS values of RF, BF, and ST on the contralateral side, and group B was significantly higher than group C. The RMS value of contralateral side of VM in group A was significantly larger than that in group C (Table 6; Figure 7).

**TABLE 6:** SEMG values during ankle dorsiflexion training.

	Groups	Mean ± SD	Groups		P-value
Affected side -RF	A	73.5 ± 74.03	A	B	0.524
	B	53.79 ± 35	A	C	0.739
	C	83.23 ± 112.88	B	C	0.249
Contralateral side-RF	A	47.17 ± 30.8	A	B	0.115
	B	80.37 ± 87.9	A	C	0.936
	C	45.61 ± 37.93	B	C	**0.046**
Affected side -VL	A	109.87 ± 107.15	A	B	0.975
	B	102.26 ± 42.8	A	C	0.403
	C	305.52 ± 1,010.29	B	C	0.32
Contralateral side -VL	A	144.67 ± 84.18	A	B	0.388
	B	110.07 ± 64.73	A	C	0.624
	C	126.2 ± 143.91	B	C	0.624
Affected side -VM	A	53.85 ± 34.8	A	B	0.141
	B	76.24 ± 47.07	A	C	0.8
	C	57.44 ± 41.33	B	C	0.133
Contralateral side -VM	A	116.9 ± 82.15	A	B	**0.016**
	B	51.34 ± 48.33	A	C	0.115
	C	77.12 ± 84.58	B	C	0.239
Affected side -BF	A	18.17 ± 22.04	A	B	0.935
	B	48.49 ± 63.91	A	C	0.394
	C	316.47 ± 1,517.49	B	C	0.381
Contralateral side -BF	A	32.34 ± 30.36	A	B	0.523
	B	37.54 ± 27.98	A	C	0.157
	C	21.41 ± 12.21	B	C	**0.019**
Affected side -ST	A	34.32 ± 27.06	A	B	0.678
	B	41.53 ± 44.89	A	C	0.909
	C	36.19 ± 57.71	B	C	0.709
Contralateral side -ST	A	27.15 ± 24.15	A	B	**0.013**
	B	69.93 ± 76.6	A	C	0.652
	C	20.01 ± 18.16	B	C	**0.001**

SEMG, surface electromyography; RF, rectus femoris; VM, vastus medialis; VL, vastus lateralis; BF, biceps femoris; ST, semitendinosus.

That the bold values indicates the statistical significance was established.

## 4 Discussion

This study explored the muscles’ SEMG characteristics of patients with ACL injuries in different rehabilitation phases. The results showed that the maximum muscle strength has a significant tendency to decrease, and the attenuation of each muscle during different movements was also different. However, muscle strength recovery in BF and ST are significantly poorer. Although muscle strength and symmetry of the bilateral lower extremities play an important role in patient recovery, muscle atrophy is common in patients who require local fixation. Factors related to muscle size and strength loss after ACLR remain unclear. The decrease in the maximum muscle strength of the BF and ST may be due to the influence of the operation. Furthermore, some studies have been conducted on age, preoperative and postoperative activity levels, the time elapsed since surgery, ACL grafts, rehabilitation programs, concurrent operative procedures, and other methods have been used to define muscle size and function. Muscle atrophy appears to occur regardless of the type of graft used to reconstruct the ACL and often occurs bilaterally (Papannagari et al., 2006). The pattern of muscle strength decline is not the same for different muscles, Li et al. showed that Among the VL, VM, and RF, the recovery rate of VL is the slowest (Li et al., 2020). Kim et al. also found significant differences in the recovery rates of extensor muscles and flexor muscles of knee (Kim et al., 2015).

Changes in the biomechanics of the lower extremities are also important factors that lead to muscle changes. Surgery cannot completely achieve the anatomical reduction of the ACL, which can lead to a change in torque. The symmetry of both legs changes due to the change in torque, especially during contralateral movements. Numerous studies have evaluated the effect of common musculoskeletal impairments after ACLR on lower-extremity movement asymmetries. Some studies have suggested that patients with high quadriceps strength symmetry demonstrated lower limb movement symmetry and greater functional performance than those with low quadriceps strength symmetry (Lewek et al., 2002; Palmieri-Smith and Lepley, 2015). Moreover, many studies have found that the biomechanical characteristics of both lower extremities are still inconsistent after ACLR. Roewer et al. (2011) reported asymmetric knee angles and moments after ACLR in participants with symmetrical quadriceps strength.

Additionally, Curran et al. (2018) reported that although quadriceps strength improved after reconstruction, there were still asymmetric biomechanical parameters of the knee. Shi et al. (2019) reported that the isometric quadriceps strength of the injured leg was significantly lower than that of the uninjured leg. Knee flexion angles and knee extension moments were smaller in the injured leg than in the uninjured leg during the loading response and mid-stance phases. Asymmetry in the maximum knee flexion angle during the loading response and mid-stance phases was significantly correlated with asymmetry in the isometric quadriceps strength. Isometric quadriceps strength was also significantly correlated with the asymmetry in the peak knee extension moment during the midstance phase. The authors believed that individuals receiving ACLR demonstrate asymmetry in knee movement in the sagittal plane. Isometric quadriceps strength asymmetry was significantly correlated with the asymmetry in knee flexion angles during the early stance phase and with the knee extension moments during the midstance phase. Palmieri-Smith and Lepley (2015) also suggested that decreased QF strength is associated with altered trunk biomechanics of the lower extremity and sagittal plane during landing tasks after ACLR. However, the relationship between QF muscle strength and lateral trunk control after ACLR during landing has not been examined. Furthermore, weakness in the hip abductor (HA) muscles is often associated with a dynamic valgus of the knee during landing activities, a pattern of motion associated with both primary and secondary risks of ACL injury (Petersen et al., 2014). Therefore, rehabilitation programs should emphasize eccentric exercise to improve neuromuscular control.

This study showed that different muscles exhibited an obvious decrease in muscle activation under different exercise conditions, which closely related to the patient’s sports ability. For example, the muscle strength of the contralateral side of the RF, the contralateral side of the VM, and the affected side of the BF changed during normal walking and that of the affected side of the RF, the contralateral side of the RF, and the affected side of the VL changed during a fast walking movement. ACL structures also contain mechanoreceptors, injuries of which cause afferent block-mediated dysregulation of motor control of the spine and spinal cord, directly affecting neuromuscular control of the knee. ACL injury can cause changes in biomechanical function, proprioceptive function, and strategy of motor control (Van Melick et al., 2016). Begalle et al. (2012) quantified the activity level of VM, VL, MH, and BF muscles by SEMG. In exercises such as the single-limb squat, the transverse-lunge, lateral-lunge, etc, the extensor and flexor muscles of the knee show a marked difference. Therefore, the muscle training method must refer to the target motion modes and evaluate the quality of movement. The focus on quality evaluation of movement is not only a risk factor for a second ACL injury, but also an important factor for motor rehabilitation.

## 5 Limitation

Only 60 patients participated in this study, and the limited sample size reduced the informative value of the study. The lack of follow-up also reduces the representativeness of the results. Therefore, we will continue to expand the sample size of this study, achieve long-term follow-up of patients, and provide more evidence to validate the results.

## 6 Conclusion

Overall, the muscle activation of various muscles differ in several movements. Therefore, according to the test results under different exercise conditions, we may find which muscle requires more training and improves the functional status of patients after surgery. We suggest developing a rehabilitation program based on target orientation. At different phases of rehabilitation, testing the activation of muscles under different training tasks, and the training tasks and the intensity of the training tasks are selected based on this. We speculate that there will be better rehabilitation.

## Data Availability

The raw data supporting the conclusions of this article will be made available by the authors, without undue reservation.
